# Effects of fresh, aged and cooked garlic extracts on short- and long-term memory in diabetic rats

**Published:** 2013

**Authors:** Alireza Sarkaki, Saeed Valipour Chehardacheric, Yaghoub Farbood, Seyed Mohammad Taghi Mansouri, Bahareh Naghizadeh, Effat Basirian

**Affiliations:** 1 *Physiology Research Center (PRC), Medicinal Plant Research Center, Ahvaz Jundishpur University of Medical Sciences, Ahvaz, I.R. Iran*; 2 *Department of Biology, Faculty of Sciences, Islamic Azad University, Izeh branch, Khouzestan, I.R. Iran *; 3 *Department of Physiology, Medicine Faculty, Physiology Research Center (PRC), Ahvaz Jundishpur University of Medical Sciences, Ahvaz, I.R. Iran*; 4 *Department of Pharmacology, Medicine Faculty, Physiology Research Center (PRC), Ahvaz Jundishpur University of Medical Sciences, Ahvaz, I.R. Iran *; 5 *Physiology Research Center, Ahvaz Jundishapur University of Medical Sciences, Ahvaz, I.R. Iran*

**Keywords:** Garlic, Hyperglycemia, Memory, Rats, Streptozotocin

## Abstract

**Objective:** The present study was hypothesized to investigate the beneficial effects of fresh, aged, and cooked garlic extracts on blood glucose and memory of diabetic rats induced by streptozocine (STZ).

**Material and Methods:** Diabetes was induced by an intraperitoneal injection of STZ (60 mg/kg body weight). An oral dose of 1000 mg/kg of each garlic extract was given daily for 4 weeks after diabetes induction. Five days after STZ injection, five groups were formed: Control (intact) rats (Cont) + Vehicle of garlic extract (normal saline) (Veh), STZ + Veh, STZ + Fresh (row) garlic (FG), STZ + Aged garlic (AG), and STZ + cooked (boiled) garlic (CG). In order to assess the passive avoidance memory, rats were gently placed on the wooden platform, and latency to step-down (SDL) was recorded as initial phase, after then a light electrical shock [0.3 mA, 3 sec, Alternative current (AC)] was delivered to their foot paw. The retrieval tests were done for short- and long-term memories, respectively. Blood glucose was assayed by glucometer before and after treatment with STZ and garlic extracts.

**Results: **Hyperglycemia induced by STZ decreased short-term memory in both diabetic males and females rats significantly compared with the controls (p<0.001 and p<0.01). Fresh and cooked but not aged garlic extracts decreased blood glucose in diabetic males and increased memory in both diabetic male and female rats significantly (p<0.05 and p<0.01).

**Conclusions:** STZ causes elevation of the blood glucose and resulted in memory deficits, possibly viafree radicals production in brain tissue. Garlic has some bioactive chemicals including allicin and sulfur compound (OSC) which could lower the blood glucose during chronic hyperglycemia, inhibit free radicals production in brain, and improve short-term (but not long-term) memory.

## Introduction

Central nervous system complications including cognitive impairment are an early manifestation of diabetes mellitus and are also evident in animal models (Idan-Feldman et al., 2011 ▶). Memory impairment induced by streptozotocin (STZ) in rats is a consequence of changes in CNS that are secondary to chronic hyperglycemia, impaired oxidative stress, cholinergic dysfunction, and changes in glucagon-like peptide (GLP). Treatment with antihyperglycemics, antioxidants, and cholinergic agonists are reported to produce beneficial effect in this model (Bhutada et al., 2011 ▶).

Abnormal regulation of glucose and impaired carbohydrate utilization that result from a defective or deficient insulin are the key pathogenic events in type 2 diabetes mellitus (Mahmoud et al., 2012 ▶). Studies indicate that STZ induces deficit in learning/memory, decrease in synaptophysin (SYP) expression, and degeneration in synaptic structures (Hou et al., 2012 ▶). Hyperglycemia and perturbed insulin signaling have been proposed as pathogenic factors contributing to AD. Recent findings strengthen the case for insulin as therapy for AD afflicted individuals with or without diabetes (Subramanian and John 2012 ▶). Growing evidence suggests that type 2 diabetes mellitus (DM) is associated with age-dependent Alzheimer's disease (AD), the latter of which has even been considered as type 3 diabetes. Several physiopathological features including hyperglycemia, oxidative stress, and dysfunctional insulin signaling relate DM to AD (Chen et al., 2011 ▶). Diabetic metabolic disorder contributes to beta-amyloid protein (A-beta) generation (Cai et al., 2011 ▶). Nutritional therapy is a challenging but necessary dimension in the management of diabetes and neurodegenerative changes associated with it (Kumar et al., 2011 ▶). Herbs and spices have a traditional history of use, with strong roles in cultural heritage, and in the appreciation of food and its links to health. Demonstrating the benefits of foods by scientific means remains a challenge, particularly when compared with standards applied for assessing pharmaceutical agents. Pharmaceuticals are small-molecular-weight compounds consumed in a purified and concentrated form. Food is eaten in combinations and in relatively large and unmeasured quantities under highly socialized conditions (Tapsell et al., 2006 ▶). Garlic (Allium sativum) is regarded as both a food and a medicinal herb. Increasing attention has been focused on the biological functions and health benefits of garlic as a potentially major dietary component (Haider et al., 2008 ▶). 

The medicinal benefits of Allium vegetables, especially garlic, have been noted throughout recorded history. The known health benefits of Allium vegetables and their constituents include cardiovascular protective effects, stimulation of immune function, reduction of blood glucose level, radioprotection, improvement of memory loss, protection against microbial, viral and fungal infections, as well as anticancer effects. Population-based case control studies have suggested an inverse correlation between dietary intake of Allium vegetables and the risk of different types of cancers (Herman-Antosiewicz et al., 2007 ▶). 

From ancient times, garlic (Allium sativum) has been used to treat several diseases. Recent findings suggest thataged garlic extract (AGE) may be a therapeutic agent for AD because of its antioxidant and A-beta lowering properties. Byaging of garlic, some adverse reactions of garlic can be eliminated. To date, the molecular properties of AGE have been sparsely studied *in vitro* or *in vivo* (Ray et al., 2011 ▶).

Feeding aged garlic extract prevented deterioration of hippocampal based memory tasks in 7-month-old Tg2576 mice model showing slow plaque development with a predominance of A-beta (1-40) which may correlate with the mild cognitive impairment (MCI) stage of AD, suggesting that aged garlic extract has a potential for preventing AD progression (Chauhan and Sandoval 2007 ▶). 

The antioxidative activity and ameliorative effects on memory impairment by sulfur-containing compounds which occur in Allium vegetables such as onion and garlic were investigated (Nishimura et al., 2006 ▶). Risk factors for cardiovascular diseases, including high cholesterol, high homocysteine, hypertension, and inflammation, increase the risk of dementia, including its most common form, Alzheimer's disease (AD) (Borek 2006 ▶).

Administration of rutin and garlic oil before global cerebral ischemia markedly reduced cerebral infarct size and attenuated impairment in short-term memory and motor coordination (Gupta et al., 2003 ▶). On the other hand, studies suggest the possibility that AGE prevents physiological aging and age-related memory disorders in human (Moriguchi et al., 1996 ▶). The acute toxicity test of garlic extract was studied in Wistar rats. The LD50 values of garlic extract by p.p., i.p. and S.C. administration were estimated over 30 ml/kg, respectively, in male and female rats and no specific signs due to garlic extract were observed in survivals for 7 days (Nakagawa et al., 1984 ▶). The influence of garlic extract on the chronic toxicity test was examined orally in Wistar rats for 6 months. There were no toxic symptoms due to garlic extract even at dose level of 2000 mg/kg for 5 times a week during 6 months (Sumiyoshi et al., 1984 ▶). 

Based on the current literatures and our knowledge about different consumption types of garlic as nutrient and medicine, we proposed that different forms of garlic extract consumption may have different effects on memory in diabetic rats. Therefore, the aim of this study was to assess the effects of three types of garlic (fresh, aged, and boiled) as hydroalcoholic extracts consumption on blood glucose and cognition in STZ-induced diabetic rats as assessed by passive avoidance task.

## Materials and Methods

Seventy adult male and female Wistar rats (220±30 g) were used in this study. They housed in standard cages as 7 in a cage separated according to sex under controlled room temperature (20±2 °C), humidity (50-55%) and 12:12h light/dark cycle and free access to food and water ad libitum. All experiments were controlled by the Local Ethics Committee for the Purpose of Control and Supervision of Experiments on Laboratory Animals. Ten days after arriving in the laboratory, the rats were divided into two main male and female groups. Then, rats in each group were randomly divided into five equal groups of 7 as: Main male sub-groups and main female sub-groups. Sub-groups were as following: 

1. Groups 1 and 2: Control males and females (Cont+Veh), intact rats received 5 ml/kg normal saline for 28 days, orally. 

2. Groups 3 and 4: Male STZ+Veh and female STZ+Veh. Diabetes was induced by an intraperitoneal streptozotocin injection (60 mg/kg) (Idan-Feldman et al., 2011 ▶) and from 5^th^ day (after confirming the hyperglycemia by glucometer with at least 250 mg/dl as fasting serum glucose) received the same volume of normal saline p.o. for 28 days as garlic extract vehicle. 

3. Groups 5 and 6: Male STZ+FG and female STZ+FG. They received 60 mg/kg STZ, i.p. and from 5^th^ day (after hyperglycemia confirmation by glucometer with at least 250 mg/dl as fasting serum glucose) received 1000 mg/kg/5ml fresh garlic extract dissolved in normal saline p.o. for 28 days (Morihara et al., 2002 ▶; Taleb-Senouci et al., 2012 ▶). 

4. Groups 7 and 8: Male STZ+AG and female STZ+AG. They received 60 mg/kg STZ, i.p. and from 5^th^ day (after hyperglycemia confirmation by glucometer with at least 250 mg/dl as fasting serum glucose) received 1000 mg/kg/5ml aged garlic extract dissolved in normal saline p.o. for 28 days. 

5. Groups 9 and 10: Male STZ+CG and female STZ+CG. They received 60 mg/kg STZ, I.P. and from 5^th^ day (after hyperglycemia confirmation by glucometer with at least 250 mg/dl as fasting serum glucose) received 1000 mg/kg/5ml cooked (boild) garlic extract dissolved in normal saline p.o. for 28 days. Blood glucose was measured in control, STZ, and treated diabetic rats with different forms of garlic extracts. 

**Garlic extracts preparation**

Garlic vegetable (*Allium sativum *L.*)* belonging to Alliaceae plants family was purchased from Ramhormoz garlic farms, Khouzestan, Iran and was stored in room temperature (one meter above the ground by a thin rope) for at least 12 months to produce the aged garlic (Efendy et al., 1997 ▶). The fresh garlic was cooked in boiled water for at least one hour to produce the cooked garlic (Cavagnaro and Galmarini 2012 ▶; Gorinstein et al., 2006 ▶). Their peels were removed and thin sliced, air dried in shade for one week and milled to fine powder (electric mill, Panasonic Co. Japan). The garlic powder was macerated in 80% ethanol for 72 hours at room temperature. The ethanol extract evaporated (Rotary Ovaporator, Heidolph Co. Germany) to remove ethanol and garlic extract was obtained as a lyophilized powder (yield 25-30%) (Jalal et al., 2007 ▶). 

**Passive avoidance task**

This procedure was similar to that previously described (Saadipour et al., 2009 ▶). Briefly, at first day of experiment, rats were acclimated to the acquisition chamber. At the second day, the rats were gently placed on the wooden platform and latency to stepdown (SDL) was recorded as the initial phase. When all four paws touched the grid, a low level electric shock (0.3 mA, 3 sec) was delivered. On days 1, 3, 7, and 14 aftershock delivery to the rats, step-down latencies were measured (maximum 300 sec.) while no shock was applied. 

**Statistical analysis**

Data were expressed as mean±SEM for male and female rats separately. Step-down latencies at 1^st^, 3^rd^, 7^th^, and 14^th^ day of retention trials in each sex were analyzed by one-way ANOVA, followed by LSD post-hoc tests. The statistical significance was considered as p<0.05. 

## Results

**Figure 1:**
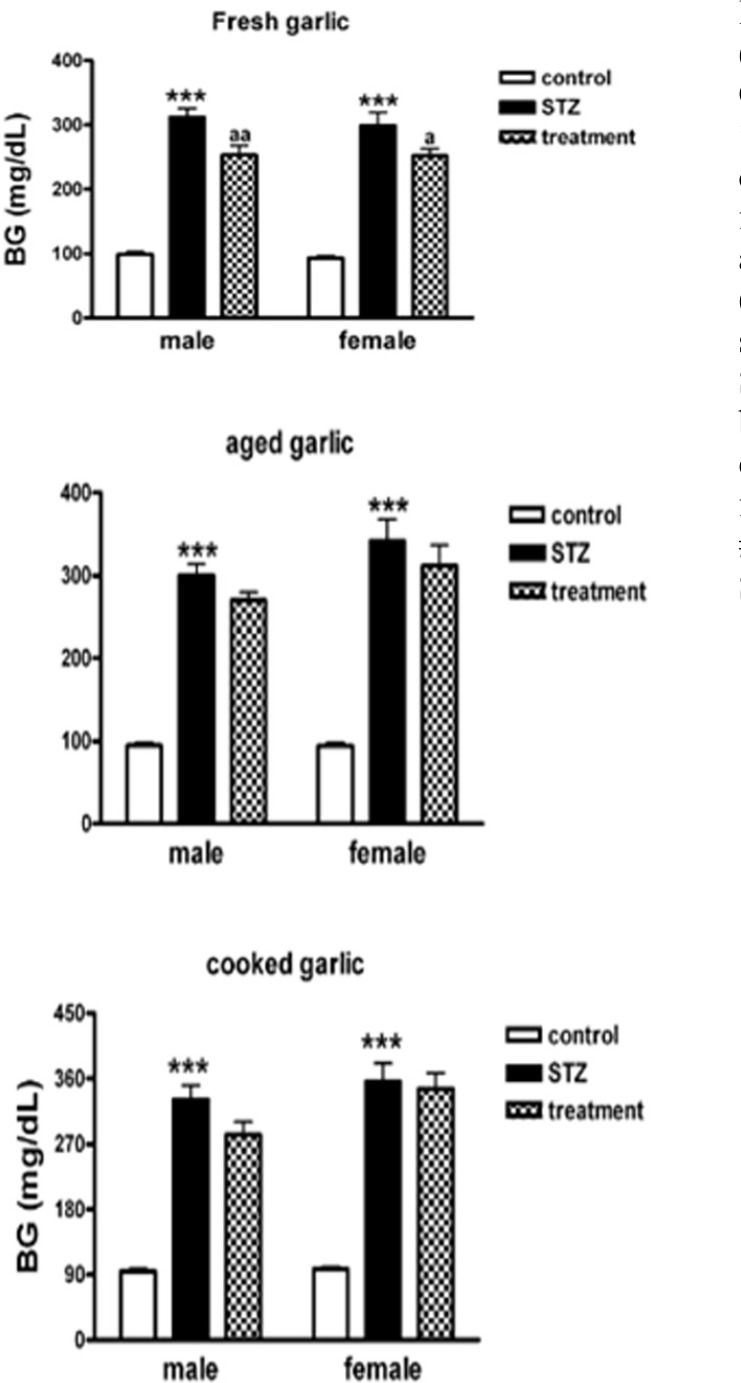
Mean±SEM. of blood glucose (BG) in control, diabetic (STZ), treated diabetic rats with fresh, aged, and cooked garlic (n=10, one way ANOVA followed by LSD post-hoc test was done for each sex group separately. Symbole * used for highlighting diabetic groups (STZ) in each sex vs. control, a used for difference between treated groups with garlic vs. diabetic (STZ) group in each sex

Blood glucose increased significantly after administration of STZ in male and female rats compared with control groups (p<0.001). Treatment of the diabetic male and female rats with fresh garlic extract lowered blood glucose significantly (p<0.05), but not aged and cooked extracts (Figure 1 left, middle and right panels).

**Figure 2:**
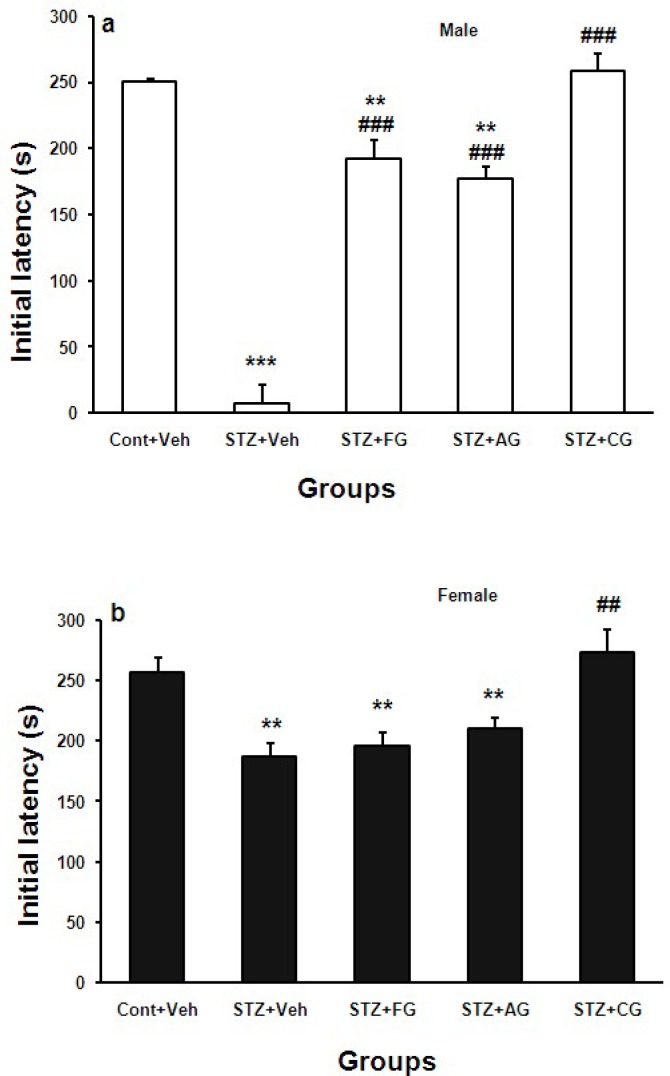
Mean±SEM. of Initial latency (IL) (s) in Cont+Veh, STZ+Veh, STZ+FG, STZ+AG, and STZ+CG male (a) and female (b) rats groups (n=7, one way ANOWA followed by LSD post-hoc test was done for each sex groups separately. STZ= diabetic, FG= fresh garlic extract, AG= aged garlic extract, CG= cooked garlic extract. Symboles * used for highlighting treated groups vs. Cont+Veh in each sex, # used for highlighting treated groups vs. STZ+Veh in each sex

As shown in Figure 2 (panels a and b), initial latency (IL) (seconds) to leave the wooden platform and touch the grid before exposing to electrical shock to rats’ foot paw was lower in diabetic male and female (STZ+Veh) significantly compared with Cont+Veh groups (***p<0.001 for males and **p<0.01 for females). IL was lower in diabetic males in comparison with diabetic females (STZ+Veh). Consumption of fresh and aged garlic extracts in diabetic males (STZ+FG and STZ+AG) increased IL significantly (##p<0.01 for STZ+FG and STZ+AG vs. male STZ+Veh, respectively) but not in diabetic females. Cooked garlic extract increased IL in both diabetic male and female groups (STZ+CG) (###p<0.001 and ##p<0.01 for male and female groups vs. STZ+Veh, respectively (Figures 2a and b).

**Figure 3:**
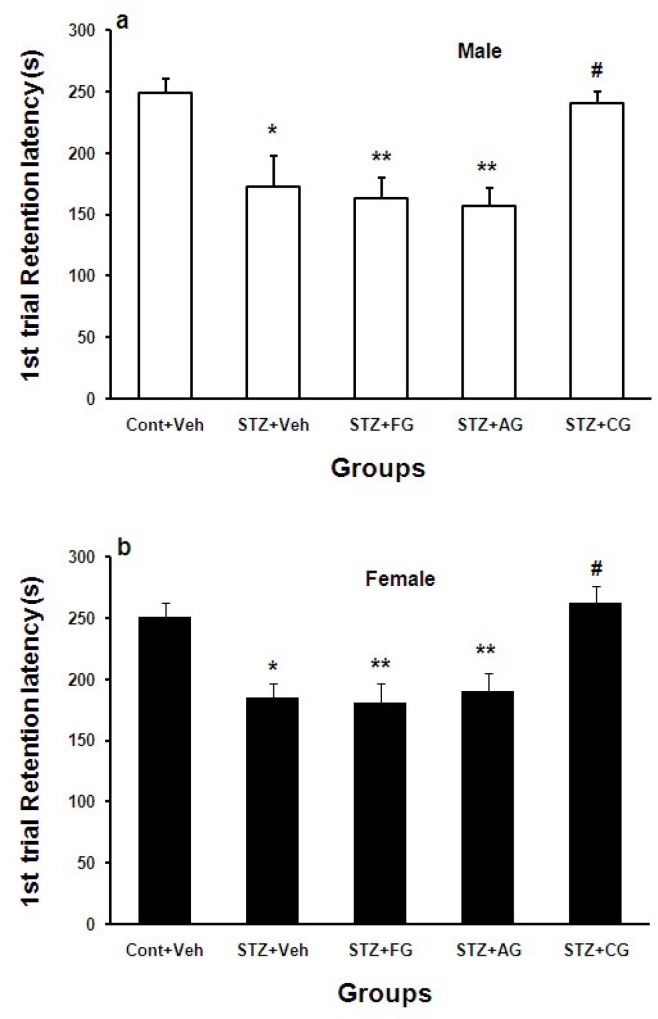
Mean±SEM. of step-down latency (SDL) during 1^st^ day after shock delivery to foot paw in Cont+Veh, STZ+Veh, STZ+FG, STZ+AG, and STZ+CG male (a) and female (b) rats groups (n=7, one way ANOWA followed by LSD post-hoc test was done for each sex groups separately. STZ= diabetic, FG= fresh garlic extract, AG= aged garlic extract, CG= cooked garlic extract). Symboles * used for highlighting treated groups vs. Cont+Veh in each sex, # used for highlighting treated diabetic rats with cooked garlic extract vs. STZ+Veh in each sex

Retention test on 1^st^ day after shock delivery to foot paw (short-term memory) showed that step-down latency (SDL) of males and females was decreased significantly during memory trials in STZ+Veh groups when compared with Cont+Veh groups (*p<0.05 for diabetic males and females (STZ+Veh) vs. Cont+Veh group, respectively). SDL was increased significantly in diabetic males and females treated with cooked garlic extract (STZ+CG) compared with STZ+Veh groups (#p<0.05 for male and female in STZ+CG groups vs. STZ+Veh. SDL did not change in diabetic males and females treated with fresh and aged extracts (Figures 3a and b). 

**Figure 4:**
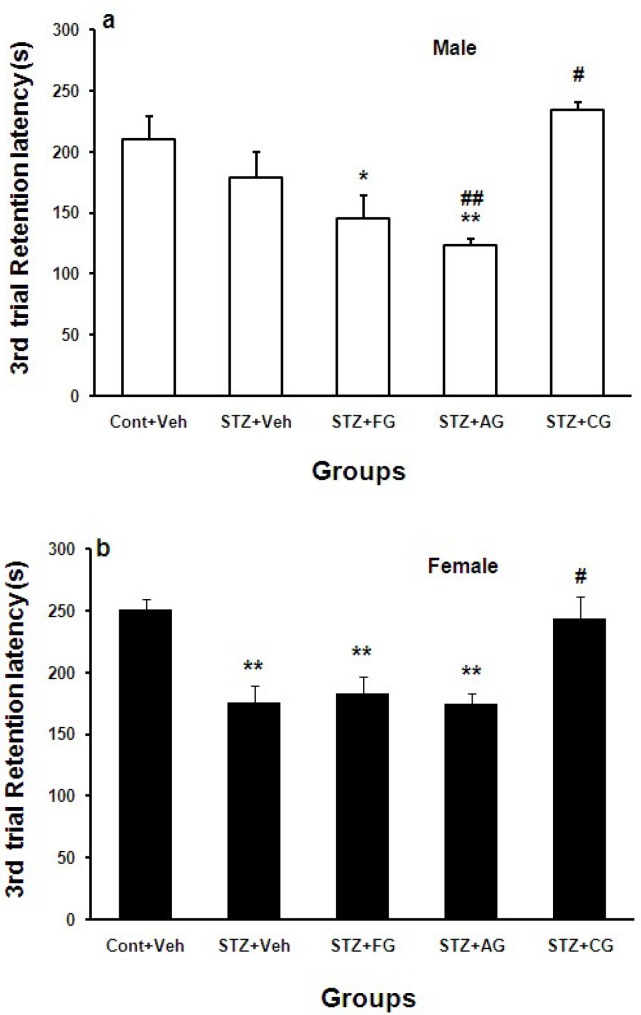
Mean±SEM. of step down latency (SDL) (s) during 3^rd^ day after shock delivery to foot paw in Cont+Veh, STZ+Veh, STZ+FG, STZ+AG, and STZ+CG male (a) and female (b) rats groups (n=7, one way ANOWA followed by LSD post-hoc test was done for each sex groups separately. STZ= diabetic, FG= fresh garlic extract, AG= aged garlic extract, CG= cooked garlic extract). Symboles * used for highlighting treated groups vs. Cont+Veh in each sex, # used for highlighting treated groups vs. STZ+Veh in each sex

Retention test during memory trials at 3^rd ^day after shock delivery to foot paw (mid-term memory) showed that step-down latency (SDL) was decreased significantly in diabetic females (STZ+Veh) group compared with Cont+Veh (**p<0.01). SDL was also decreased in diabetic males but this was not significant. SDL was increased only in diabetic males and females treated with cooked garlic extract (STZ+CG) significantly compared with STZ+Veh groups (#p<0.05). SDL waslowerin diabetic males receiving aged garlic extract (##p<0.01) (Figures 4a and b). 

**Figure 5:**
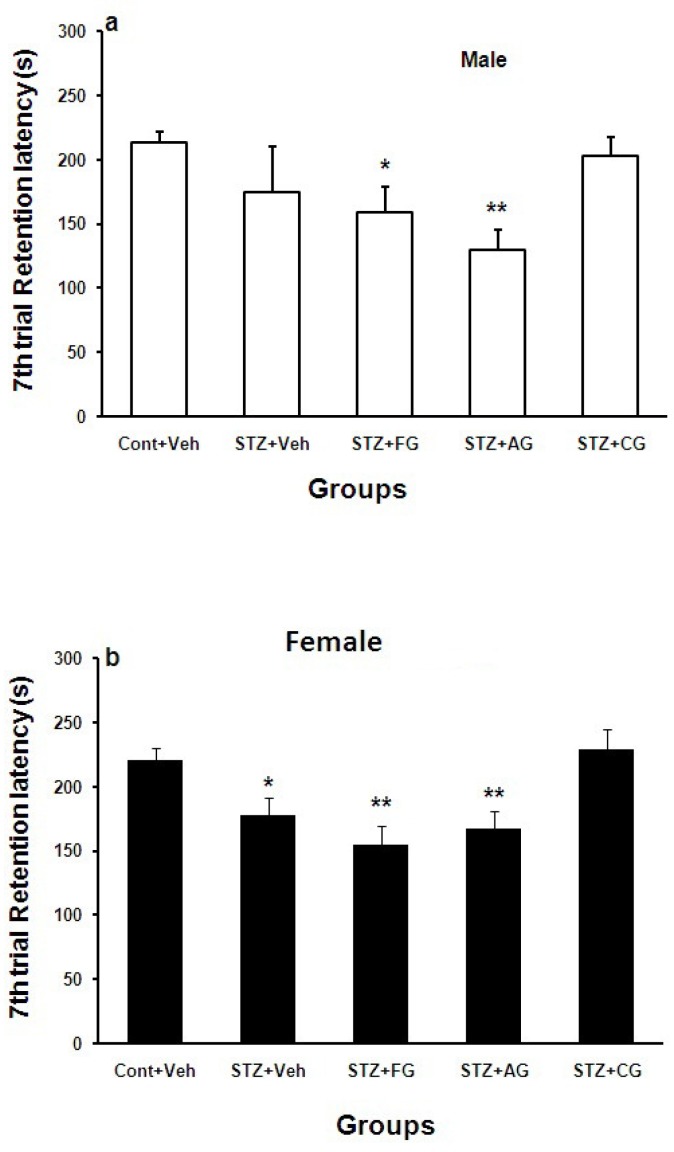
Mean±SEM. of step down latency (SDL) during 7^th^ day after shock delivery to foot paw in Cont+Veh, STZ+Veh, STZ+FG, STZ+AG, and STZ+CG males (a) and females (b) rats groups (n=7, one way ANOWA followed by LSD post-hoc test was done for each sex groups separately. STZ= diabetic, FG= fresh garlic extract, AG= aged garlic extract, CG= cooked garlic extract). Symboles * used for highlighting groups vs. Cont+Veh in each sex

Retention test on 7^th^ day after shock delivery to foot paw (long-term memory) showed that step-down latency (SDL) in diabetic females was significantly lower (*p<0.05) but not in diabetic males compared with cont+Veh groups. SDL didn’t change in treated males and females with garlic extract (Figures 5a and b). 

**Figure 6:**
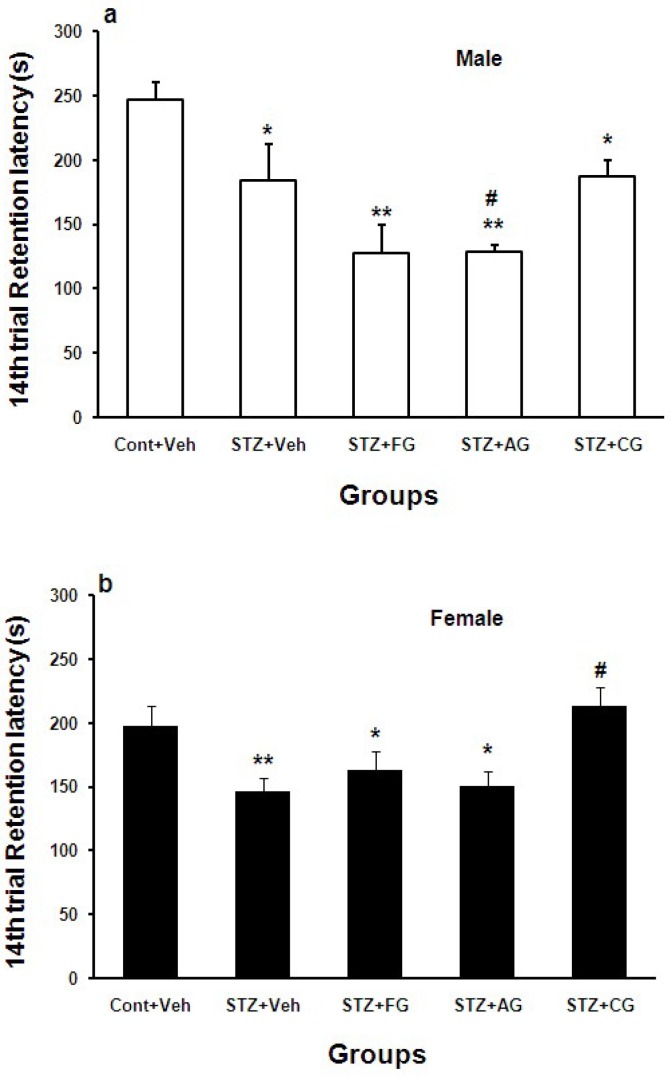
Mean±SEM. of step down latency (SDL) during 14^th^ day after shock delivery to foot paw in Cont+Veh, STZ+Veh, STZ+FG, STZ+AG, and STZ+CG males (a) and females (b) rats groups (n=7, one way ANOWA followed by LSD post-hoc test was done for each sex groups separately). STZ= diabetic, FG= fresh garlic extract, AG= aged garlic extract, CG= cooked garlic extract. Symboles * used for highlighting groups vs. Cont+Veh in each sex, # used for highlighting treated groups vs. STZ+Veh in each sex

Retention test on 14^th^ day after shock delivery to foot paw (long-term memory) showed that step-down latency (SDL) in diabetic males and females (STZ+Veh) was significantly lower than control males and females (Cont+Veh) (*p<0.05 and **p<0.01 for males and females, respectively). It increased significantly in diabetic females treated with cooked garlic extract (#p<0.05) (Figures 6a and b).

## Discussion

In this work, we investigated the effects of three forms of garlic consumption by different nutritional cultures on cognition. In overall, blood glucose (BG) was increased significantly in STZ-treated males and females. Treatment of diabetic rats with fresh garlic extract (not aged and cooked) decreased BG significantly (Figure 1). The initial latency (IL) was significantly shorter in diabetic males and females (STZ+Veh) groups than control males and females (Cont+Veh) (Figures 2a and b). Step-down latency (SDL) during short-term memory (1^st^ and 3^rd ^days after shock delivery to rats) decreased in diabetic males and females (Figures 3 and 4). SDL during short-term memory increased significantly in diabetic males and females treated with cooked garlic extract (STZ+CG). While consumption of the fresh and aged garlic extracts decreased short- and long-term memories in males and females of diabetic groups significantly (Figures 3 and 4). Our findings in the current study showed that 4 weeks administration of fresh garlic extract to diabetic male and female rats also lowered LDL, cholesterol, VLDL, and increase HDL significantly (data has not been shown here).

Hyperglycemia induced by systemic injection of STZ causes elevation of the blood glucose level and disrupts short-term memory (McNay et al., 2010 ▶; Pearson-Leary and McNay 2012 ▶). Moreover, intracerebral injection of STZ causes memory deficit associated with neurodegenerative condition in brain (Agrawal et al., 2011 ▶). Hyperglycemia at long time could disrupt the brain cognitive function maybe via accelerated free radical production and lowering the antioxidants such as GSH, vitamin C, and vitamin E while increasing MDA, NO, TNF-alpha, and IL-6 (Mahmoud et al., 2012 ▶). It is clear that hyperglycemia at first may increase memory by increasing the glucose uptake by brain tissues while at long time causes stress oxidative and thereby memory impairment in diabetic male and female rats (Figures 5 and 6). Treated diabetic male and female rats with fresh and aged garlic extracts did not reverse long-term memory but cooked garlic extract increased it in diabetic females significantly. It may be positively affected by estrogen hormone in brain as a neuroprotector (Figures 5 and 6). 

Administration of allicin, a useful biochemical of garlic, prevents learning and memory impairment; the mechanism may be due to an increase in the activity of superoxide dismutase (SOD), a reduction in the levels of malondialdehyde (MDA), and the expressions of amyloid beta_1-42 _and P38 mitogen-activated protein kinase (p38MAPK) in the brain (Belviranli et al., 2012 ▶; Li et al., 2010 ▶). Therefore, more studies are required to explore the exact mechanism of the ameliorative effects of different forms of garlic extracts against diabetic complications. Studies have shown that in the diabetic subjects, levels of glucose, glycosylated hemoglobin (HbA1c%), MDA, NO, TNF-alpha, and IL-6 were significantly increased, while serum insulin, GSH, vitamin C, and vitamin E levels were decreased (Mahmoud et al., 2012 ▶).

Chronic garlic administration to healthy subjects has been shown to enhance memory function. Evidence also shows that garlic administration in rats affects brain serotonin (5-hydroxytryptamine [5-HT]) levels. 5-HT, a neurotransmitter involved in a number of physiological functions, is also known to enhance cognitive performance (Haider et al., 2008 ▶). It is consistent with our finding that chronic administration of garlic extracts increased avoidance memory in diabetic rats (Figures 2 and 3). 

Furthermore, investigations showed that a sulfur compound (OSC), thiacremonone isolated from fresh and aged garlic, has anti-inflammatory effects (Lin et al., 2012 ▶). Moreover, the anticarcinogenic effect of Allium vegetables including fresh garlic was revealed and is attributed to OSC, which are highly effective in affording protection against cancer in animal models induced by a variety of chemical carcinogens. On the other hand, anti-angiogenic activity for garlic-derived OSC has also been documented (Herman-Antosiewicz et al., 2007 ▶). 

Investigators observed significant neuroprotective and neurorescue properties of AGE and its ingredients, from ROS (H_2_O_2_)-mediated insults to neuronal cells. Treatment with AGE was found to protect neuronal cells when they were independently co-treated with ROS. Furthermore, a novel neuropreservation effect of AGE was detected in a way that pre-treatment with AGE alone protected approximately 80% neuronal cells from ROS-mediated damage. Taken together, the neuroprotective effect of AGE can be utilized in future drug development in AD (Ray et al., 2011 ▶). In the current work, treatment of the diabetic male and female rats with aged garlic extract could not improve the short- and long-term memory. It appears that the effective substance of fresh garlic sulfur compound (OSC) may degrade in aged garlic and did not affect impaired memory in diabetic rats. 

The behavioral experiments showed that onion and also garlic extracts had highly ameliorative effect on memory impairment (Nishimura et al., 2006 ▶). Oxidative damage is a major factor in cardiovascular diseases and dementia. The risk for these diseases increases with diabetes. On the other hand, high cholesterol is also associated with hyperglycemia. It is clear that hypercholesterolemia is a risk factor to impair the memory retention. 

Studies have shown that garlic extract scavenges oxidants, increases superoxide dismutase, catalase, glutathione peroxidase, and glutathione levels and inhibits lipid peroxidation and inflammatory prostaglandins. Garlic extract reduces cholesterol synthesis. Inhibition of cholesterol, LDL oxidation, and platelet aggregation by garlic inhibits arterial plaque formation, which is important in diabetes. Garlic may also help to prevent cognitive decline by protecting neurons from A-beta neurotoxicity and apoptosis, thereby preventing ischemia- or reperfusion-related neuronal death and improving learning and memory retention (Borek 2006 ▶). 

Extracts of fresh garlic that are aged over a prolonged period to produce aged garlic extract (AGE) contain antioxidant phytochemicals that prevent oxidant damage. These include unique water-soluble organosulfur compounds, lipid-soluble organosulfur components, and flavonoids, notably allixin and selenium. Long-term extraction of garlic (up to 20 months) ages the extract, creating antioxidant properties by modifying unstable molecules with antioxidant activity, such as allicin, and increasing stable and highly bioavailable water-soluble organosulfur compounds, such as S-allylcysteine and S-allylmercaptocysteine (Borek 2001 ▶). 

On the other hand, in addition to effects of different forms of garlic, the geographical property for growing different types of garlic, and its extraction method with water or with either 50% or 100% ethanol on health is revealed (Kim et al., 2005 ▶). 

Hyperglycemia during diabetes results in cognition impairment. Fresh and aged garlic extracts improved learning in diabetic male rats alone while cooked garlic extract reversed impaired memory in both diabetic male and female rats. Cooked garlic extract may have some heated resistance bioactive chemicals such as allicin and others; those could not lower blood glucose during diabetes but reversed memory retention. Garlic may inhibit production and scavenge free radicals in brain as a neuroprotector. 
